# 130 The state of hospital epidemiologist and infection preventionist staffing in the United States

**DOI:** 10.1017/ash.2026.10542

**Published:** 2026-06-23

**Authors:** Eunji Kim, Mimi Lee, JaHyun Kang

**Affiliations:** 1 Seoul National University Hospital, Seoul National University College of Nursing; 2 Seoul National University Hospital, Seoul, Republic of Korea; 3 Seoul National University College of Nursing

## Abstract

**Background:** Clostridioides difficile infection (CDI) is a major healthcare-associated infection. Although guidelines recommend multistep diagnostic algorithms and symptom-based management with contact precautions, diarrhea assessment is subjective and adherence to diagnostic algorithms may be inconsistent in clinical practice, potentially leading to unnecessary testing and prolonged contact precautions. This study aimed to evaluate real-world practices of CDI testing, symptom assessment, and contact precautions in hospitalized patients. **Methods:** We conducted a retrospective descriptive study using electronic medical record data from January through December 2025 of adult patients (≥18 years) hospitalized for at least 3 days at a university-affiliated tertiary-care hospital in South Korea with a positive Clostridioides difficile nucleic acid amplification test (NAAT). Collected variables included patient demographics, hospital unit, toxin A/B enzyme immunoassay (EIA) results, presence of diarrhea, CDI treatment, duration and reasons for discontinuation of contact precautions, and symptom status at discontinuation. Diarrhea was defined as three or more episodes of watery or loose stool within 24 hours. Cases were classified as clinically relevant CDI if they had (1) a positive NAAT and positive toxin A/B EIA result, regardless of symptoms, or (2) a positive NAAT and negative toxin A/B EIA result with documented diarrhea, consistent with clinical practice. CDI treatment was defined as ≥10 days of oral vancomycin or metronidazole (oral or intravenous). Contact precautions was discontinued after ≥48 hours without diarrhea. **Results:** A total of 269 NAAT-positive episodes were identified in 214 patients (mean age, 63.5 ± 17.5 years), most from general wards (68.4%). Toxin A/B EIA was performed in 94.8% of episodes, with 21.2% positivity, and 60.6% were classified as clinically relevant CDI. Diarrhea was documented in 61.3% of episodes, whereas contact precautions were implemented in 95.9%, including 36.1% of episodes without diarrhea. CDI treatment was administered in 75.1% of episodes, although only 49.4% of treated episodes had documented diarrhea at treatment initiation. Contact precautions were discontinued in 94.1% of episodes, with a mean duration of 12.0 ± 11.1 days. Symptom improvement was the most common reason for discontinuation (64.8%). The mean interval between the last documented diarrhea episode and contact precaution discontinuation was 7.7 ± 30.3 days, exceeding the guideline-recommended 48-hour diarrhea-free period. **Conclusions:** Contact precautions were often implemented without documented diarrhea and discontinued later than recommended. These findings indicate limitation of symptom-based CDI management in routine practice and underscore the need for more objective, standardized symptom assessment to support appropriate diagnostic stewardship and contact precautions.

